# Biomechanical investigations on compression, expansion, and flexion of tubal occluders: a finite element analysis

**DOI:** 10.3389/fbioe.2025.1675317

**Published:** 2025-10-21

**Authors:** Quan Song, Zhuo Zhang, Xiaobao Tian, Yu Chen, Fei Gao, Zhongyou Li, Lingjun Liu, Xiaoyan Wang

**Affiliations:** ^1^ Department of Mechanical Science and Engineering, College of Architecture and Environment, Sichuan University, Chengdu, China; ^2^ Sichuan Province Biomechanical Engineering Laboratory, Sichuan University, Chengdu, China; ^3^ Chengdu Neurotrans Medical Technology Co., LTD., Chengdu, China; ^4^ Department of Biomedical Engineering, Faculty of Engineering, The Hong Kong Polytechnic University, Hong Kong, Hong Kong SAR, China; ^5^ Research Institute for Sports and Technology, The Hong Kong Polytechnic University, Hong Kong, Hong Kong SAR, China; ^6^ Key Laboratory of Birth Defects and Related Diseases of Women and Children (Sichuan University), Ministry of Education, West China Second University Hospital, Sichuan University, Chengdu, Sichuan, China; ^7^ Department of Radiology, West China Second University Hospital, Sichuan University, Chengdu, Sichuan, China; ^8^ Affiliated Hospital of Jiangnan University, Wuxi, China

**Keywords:** hydrosalpinx, occluder, biomechanics, finite element analysis (FEA), medical devices, optimized design

## Abstract

**Background:**

Hydrosalpinx significantly reduces the success rate of embryo implantation no dedicated occlusion currently exists for its treatment. This study introduces a novel shape-memory-based Fallopian tube occluder and systematically evaluates its mechanical performance across designs with varying wire densities.

**Methods:**

The proposed occluder features a mesh-based support structure with a symmetrical double-coil configuration, designed to enhance friction and reduce the risk of migration. Three geometric models were developed based on wire density (n): sparse (n = 84), standard (n = 113), and dense (n = 226). Finite element simulations were conducted to assess the mechanical response of each design during crimping, deployment, and bending.

**Results:**

In the sparse model, low filament density resulted in incomplete contact with the crimping tool, producing localized stress concentrations at the support and central regions with a maximum strain of 1.88%. The standard model demonstrated improved stress redistribution toward the connection zones and achieved a peak strain of 2.73%, providing reliable radial support while maintaining moderate compliance. The dense model, although free of dominant high-stress regions, exhibited severe localized stress (up to 1569.04 MPa) and a maximum strain of 12.73%, exceeding the superelastic recovery limit of the NiTi alloy. All three designs showed minimal axial shortening and radial recoil (<3%) after deployment, indicating limited post-deployment deformation. Load–displacement analysis revealed that increasing filament density led to higher bending stiffness and reduced flexibility.

**Conclusion:**

The sparse occluder offers high flexibility but lacks adequate structural support. In contrast, the dense design suffers from excessive deformation under compression, potentially compromising structural stability. The standard configuration provides an optimal balance between flexibility and support, making it the most promising candidate for clinical application.

## 1 Introduction

Hydrosalpinx, commonly resulting from chronic salpingitis, is characterized by the accumulation of intraluminal fluid due to impaired tubal drainage. This condition significantly compromises the success of *in vitro* fertilization and embryo transfer (IVF-ET). Compared to women without hydrosalpinx, affected patients may experience up to a 50% reduction in IVF-ET success rates, along with an increased risk of early miscarriage and ectopic pregnancy (Chen et al., 2013). Establishing a receptive endometrial environment prior to assisted reproductive procedures is therefore essential for achieving successful implantation and pregnancy following IVF-ET.

In clinical practice, hydrosalpinx is typically managed through tubal ligation or proximal tubal occlusion (Tsiami et al., 2016). Among these, proximal tubal embolization has demonstrated outcomes comparable to surgical ligation, with several notable advantages: it preserves blood supply to the tubes and ovaries, effectively prevents ectopic pregnancy following complete occlusion, eliminates the need for hospitalization, reduces cost, and allows for faster postoperative recovery. These benefits are particularly significant for patients with contraindications to laparoscopic surgery, for whom embolization serves as a preferred alternative.

Originally developed as a sterilization method, tubal embolization involves the hysteroscopic insertion of micro-occlusive devices into the proximal Fallopian tubes to achieve complete luminal blockage and long-term contraception. Two devices historically used for this purpose are Essure® and Adiana®, with Essure® being more widely adopted for managing hydrosalpinx. Reports of Adiana® use in this context remain limited (Legendre et al., 2013). Similar coil-based occlusion systems, such as the Tornado microcoil (COOK Medical, USA), are also utilized in clinical settings.

The advantages of tubal embolization are particularly evident in patients who are not suitable candidates for laparoscopy, offering a minimally invasive, effective alternative. The Essure® device consists of a 40-mm-long microcoil with a 2-mm diameter. Its outer coil, made of nitinol, anchors the device at the uterotubal junction, while the inner core contains polyethylene terephthalate (PET) fibers that trigger a localized inflammatory response. This response promotes fibrotic tissue growth, ultimately resulting in complete tubal occlusion. In 2005, Rosenfield (Mijatovic et al., 2010) reported the first successful pregnancy and live birth following proximal tubal occlusion with Essure® in a patient with hydrosalpinx. In a 2010 prospective study, (Galen et al., 2011) treated ten women with hydrosalpinx using hysteroscopic placement of the Essure® device. Tubal occlusion was confirmed in 90% of cases via hysterosalpingography. Subsequent IVF or frozen embryo transfer cycles resulted in ongoing pregnancy and live birth rates of 40% and 20%, respectively. The authors concluded that tubal occlusion is a safe and effective option for women with hydrosalpinx undergoing assisted reproduction. Further supporting evidence was provided by Horwell (2017), who evaluated Essure® in 20 patients with hydrosalpinx prior to IVF—eight with unilateral and twelve with bilateral involvement. The occlusion success rate reached 97%, and the live birth rate following IVF was 67%.

Despite its demonstrated efficacy, the use of Essure® has been associated with various adverse events. Reported complications include persistent pelvic pain, uterine or tubal perforation, device migration into the pelvic or intraperitoneal cavity, abnormal bleeding, and hypersensitivity reactions (Zurawin and Zurawin, 2011). In cases of significant abdominal pain or bleeding, surgical removal of the device is often necessary. Common procedures include salpingectomy and cornual resection, while hysterectomy is associated with higher perioperative and postoperative complication rates compared to less invasive alternatives (Chene et al., 2021). Sills et al. (2017) reported that among 3,803 women who underwent surgical removal of the Essure® device, 64.9% ultimately required hysterectomy rather than device extraction alone—highlighting the substantial surgical burden associated with its complications.

Microcoils are widely employed in clinical interventional procedures to embolize peripheral vascular lesions such as aneurysms, arteriovenous malformations, and arteriovenous fistulas. To improve occlusion efficacy in both vascular structures and Fallopian tubes, these coils are often constructed using a braided architecture and integrated with nylon fibers. However, when deployed in the Fallopian tube, braided coils can be vulnerable to lateral displacement under compressive forces. This displacement may lead to loosening of the occlusive structure, uneven load distribution, and diminished mechanical stability. A primary contributing factor is the difficulty in achieving complete apposition between the coil and luminal wall during hysteroscopic placement, often resulting in incomplete occlusion (Yuan and Bing, 2025).

Moreover, multiple coils are typically required for effective occlusion in interventional procedures, and treatment failure has been reported in the context of hydrosalpinx embolization. One study highlighted the importance of selecting coil sizes tailored to the anatomical features of the Fallopian tube, such as its length and wall thickness. To achieve satisfactory embolization, several coils are usually delivered via microcatheter, and in some cases, a secondary embolization procedure is necessary (Hong et al., 2018). Collectively, these limitations underscore the pressing need for a dedicated occlusion device specifically designed for the Fallopian tube.

To address these challenges, this study proposes a novel self-deploying, double-layer coil occlusion device engineered to enhance radial support and promote uniform stress distribution within the tubal lumen. Finite element analysis was employed to systematically model and evaluate three design variants of the device, each with a different wire density. Simulations of compression, deployment, and flexibility were conducted to compare biomechanical performance and provide mechanical validation for the proposed design.

## 2 Methods and materials

### 2.1 Principle of occluder design

Nitinol was selected as the primary material for the occlusion device in place of the conventional platinum–tungsten alloy of its shape memory properties and superelastic behavior, which are particularly advantageous for minimally invasive medical applications. Additionally, nitinol exhibits excellent biocompatibility, making it well-suited for long-term implantation (Sevcikova and Pavkova Goldbergova, 2017). An ideal Fallopian tube occlusion device must demonstrate reliable expansion, strong radial support, high axial flexibility, and minimal recoil in both radial and axial directions (Vanaei et al., 2024). To optimize these mechanical characteristics, a structural framework inspired by conventional mesh stents was adopted.

**FIGURE 1:**
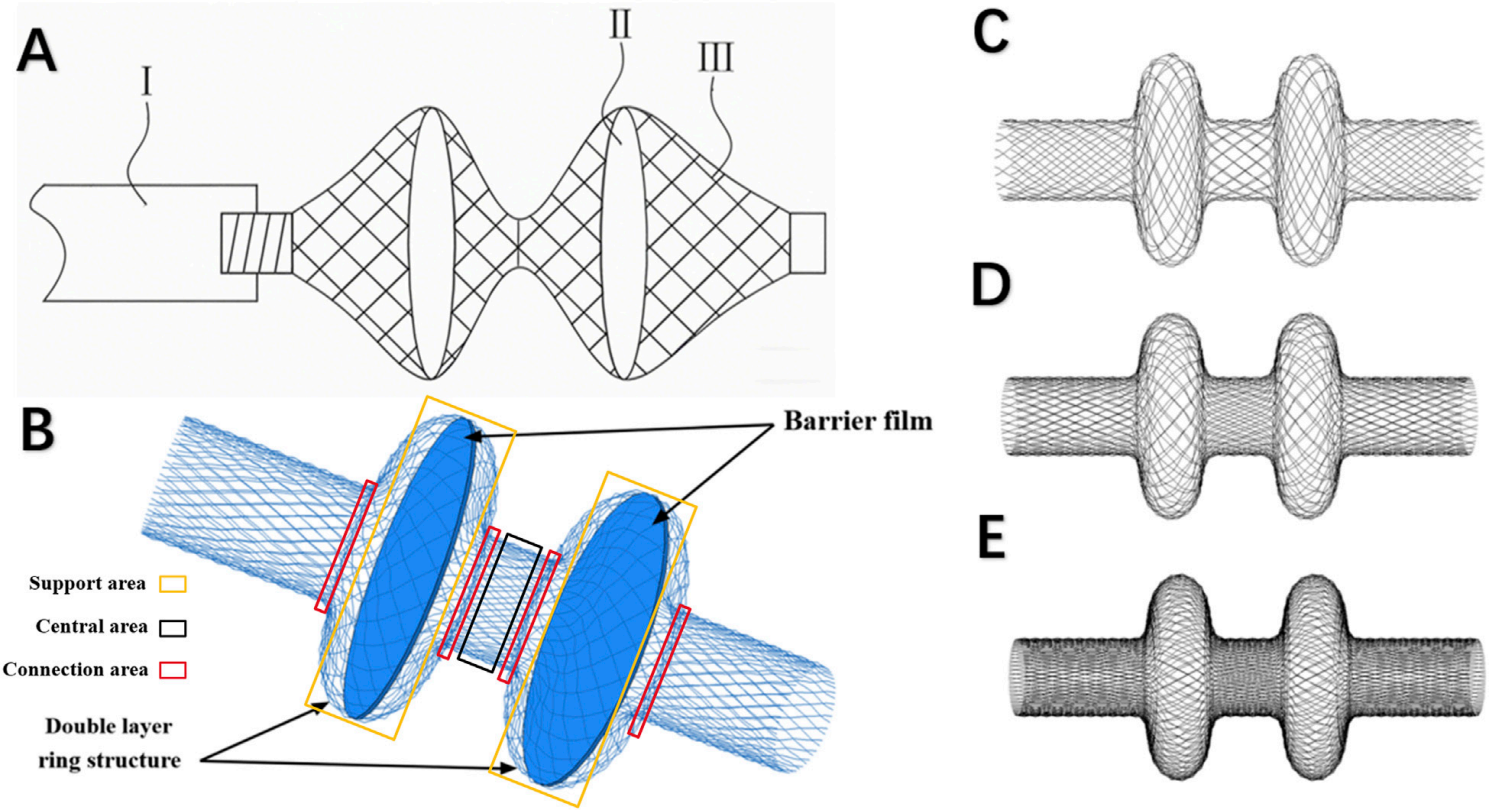
**(A)** Schematic of the two-dimensional model of the fallopian tube occlusion device (Ⅰ: Conveyor rod; Ⅱ: Barrier film; Ⅲ: Occluder); **(B)** Schematic of the double-coil structure and occlusive membrane; **(C)** Sparse model (n = 84); **(D)** Standard model (n = 113); **(E)** Dense model (n = 226).

In contrast to traditional single-coil designs—which often suffer from inadequate frictional contact with the tubal wall, leading to device migration and incomplete occlusion—a symmetric double-coil configuration is proposed. This dual-layer design significantly increases the contact surface area within the tubal lumen, thereby enhancing frictional resistance and improving positional stability. Moreover, the double-coil architecture enables more precise modulation of the radial pressure exerted on the tubal wall, contributing to both efficacy and safety. The two-dimensional and three-dimensional representations of the proposed device are illustrated in Figures 1A,B, respectively.

### 2.2 3D model and meshes

The occlusion device was modeled using SolidWorks (Dassault Systèmes, MA, United States). Based on the predefined design specifications, the primary structure was constructed using a two-dimensional profile of a standard helical coil. Initially, a single-layer coil was generated using a wire diameter of 0.02 mm. This base geometry was then replicated in a linear array and subjected to a coiling operation to produce the final three-dimensional model of the occluder. The modeling process focused specifically on evaluating how varying wire density influences the device’s mechanical performance. Accordingly, three distinct models were developed, each representing a different wire density (n): sparse (Figure 1C), standard (Figure 1D), and dense (Figure 1E). The detailed geometric parameters for all models are in Table 1. For the simulation of loading conditions, a thin-walled cylindrical compression tool was employed, measuring 30 mm in length and 8.4 mm in diameter.

**TABLE 1:** Geometric specifications of occluders (mm).

Models	Proximal/Distal diameter	Bulged-end diameter	Length	Wire diameter
Sparse (*n* = 84)	3	8	18	0.02
Standard (*n* = 113)	3	8	18	0.02
Dense (*n* = 226)	3	8	18	0.02

Beam elements were selected for mesh generation due to the relatively small wire diameter in comparison to the spacing within the support structure. This choice enables efficient parametric analysis of wire thickness while reducing the number of degrees of freedom and enhancing computational efficiency. Specifically, B31 elements, linear two-node spatial Timoshenko beam elements with linear interpolation were employed in this study. Compared with B33 and B33H elements, B31 elements can accommodate transverse shear deformation, making them suitable for a wider range of structural applications.

The compression tool was meshed using SFM3D4R elements, with element size of 0.2 mm. The total number of elements and nodes used for each component is summarized in Table 2. Mesh generation and numerical simulations were conducted using the ABAQUS/Explicit solver with double-precision computation. This approach minimizes numerical errors owing to rounding and offers greater accuracy than single-precision analysis.

**TABLE 2:** Number of mesh elements and nodes for each component.

Components	Element type	Element type	Element type
Sparse (n = 84)	B31	9770	23455
Standard (n = 113)	B31	14561	37856
Dense (n = 226)	B31	24720	68393
Compression shell	SFM3D4R	21120	44252

### 2.3 Material model

Nitinol exhibits a temperature-dependent phase transformation between its martensitic and austenitic states. At lower temperatures, the alloy stabilizes in the martensitic phase, which includes both twinned and detwinned variants. As the temperature increases, it transitions into the austenitic phase (Shaw and Kyriakides, 1997). One of the defining characteristics of nitinol is its superelasticity, which enables it to endure large, fully recoverable strains beyond the conventional elastic limit. Under external loading, the material can deform substantially; once the applied stress exceeds a critical threshold, the shape memory factor reaches 1, and further loading results in elastoplastic deformation of the martensitic phase (Lee and Kim, 2022). Upon unloading—provided the stress falls below this critical level—the material gradually returns to its original shape, with the shape memory strain reducing until full recovery is achieved. In this study, all occlusion device models were defined using a superelastic constitutive model for nitinol. As the research is currently in the preliminary verification stage, it does not involve *in vitro* experiments, and the constitutive thermomechanical cycles of nitinol materials are temporarily not considered. The material parameters used are detailed in Table 3 (Zhang et al., 2022). For simplification, the compression tool was modeled as an ideal elastic body without specific material properties.

**TABLE 3:** Material parameters of Nitinol (Zhang et al., 2022).

Parameters	Value
Austenite elastic modulus (MPa)	46728
Poisson’s ratio (austenite)	0.33
Martensite elastic modulus (MPa)	25199
Poisson’s ratio (martensite)	0.33
Stress–temperature coefficient (loading)	4.50
Start of phase transformation (loading) (MPa)	358.20
End of phase transformation (loading) (MPa)	437.80
Stress–temperature coefficient (unloading)	4.50
Start of phase transformation (unloading) (MPa)	124.50
End of phase transformation (unloading) (MPa)	17.75
Start of phase transformation under compression	537.30
Transformation strain (%)	4.26
Reference temperature (°C)	0
Density (g/cm^3^)	6.5

### 2.4 Boundary conditions

**FIGURE 2:**
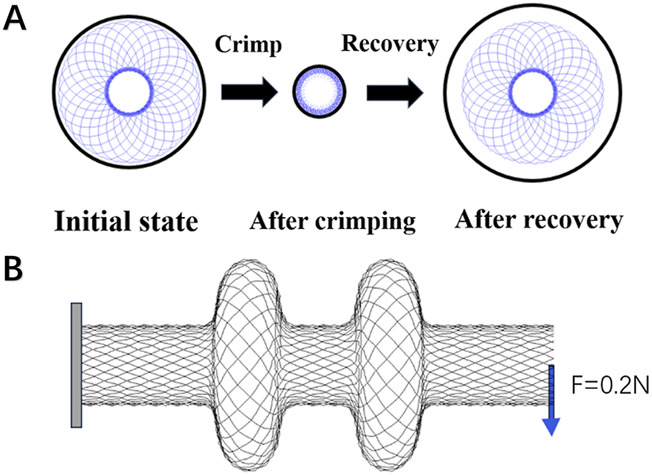
**(A)** Crimping and self-expansion; **(B)** Flexibility test. One end of the occluder is fixed, and a force of 0.2 N is applied to the other end.

To prevent rigid-body motion during simulation, the central longitudinal axis of the occlusion device was coupled to a distal reference point. This reference point was constrained in both circumferential and axial directions, and similar constraints were applied to the compression tool to eliminate any undesired global translations or rotations of the system. During the crimping phase, the simulation temperature was set to 5 °C to reflect the device’s storage conditions. A radial displacement of 3.75 mm was applied to the compression tool, directed inward toward the center of the lumen, to compress the occluder and reduce its diameter to fit within the intraluminal space. Following the completion of crimping, the temperature was increased to 36.5 °C to simulate physiological conditions. At this stage, the contact between the compression tool and the device was removed, allowing the occluder to self-expand and recover its pre-crimped configuration (Figure 2A).

To assess the flexibility of the device, a cantilever-beam configuration was employed. A thin square plate (5 mm × 5 mm, thickness: 0.2 mm) was modeled at the left end of the occluder to provide a fixed support. This plate was assigned the material properties of structural steel, with an elastic modulus of 206,000 MPa, Poisson’s ratio of 0.3, and a density of 7.85 g/cm^3^. A general contact algorithm incorporating frictionless hard contact conditions was implemented to prevent self-contact or interpenetration of the device during bending deformation. At the distal end, a reference point was created and coupled to all nodes on the right side of the occluder. A concentrated force of 0.2 N was then applied to this reference point to induce bending deformation (Figure 2B).

## 3 Results

### 3.1 Compression and deployment

**FIGURE 3:**
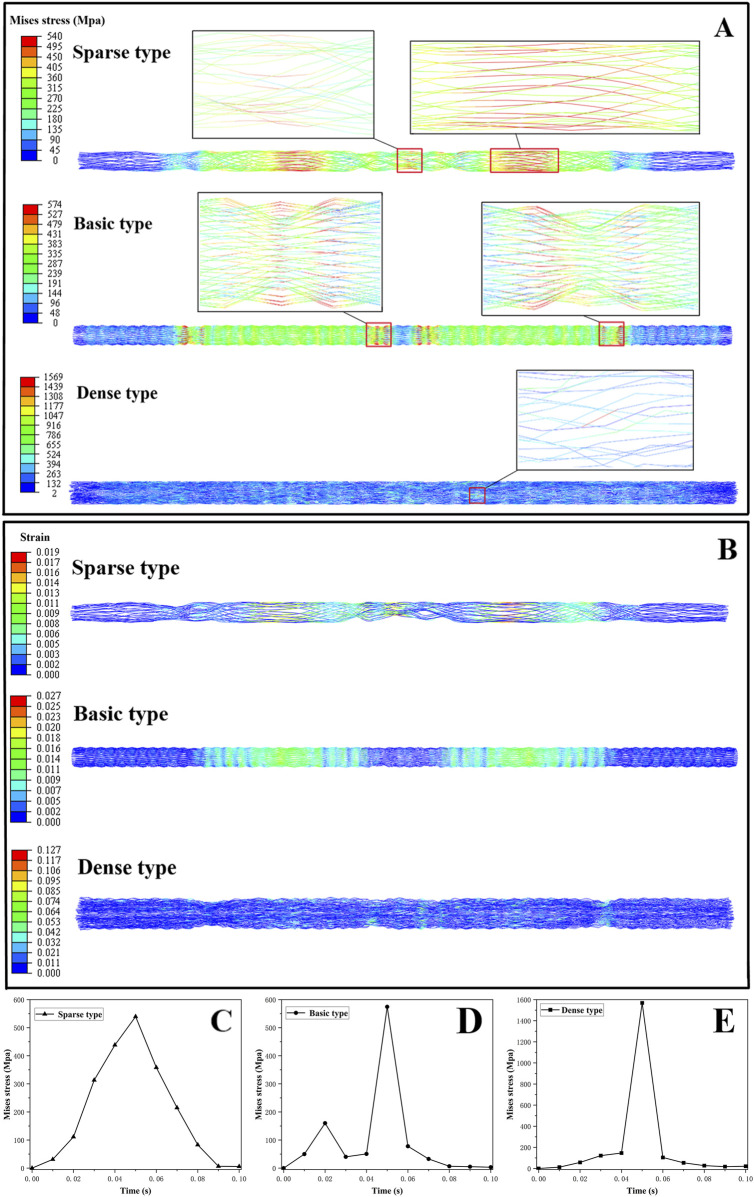
**(A)** Stress cloud map of occluder; **(B)** Strain cloud map of occluder; **(C**–**E)** stress varied with loading time for parse, standard, and dense occluders, respectively.

The maximum von Mises stresses recorded during crimping to the minimum diameter were 539.58 MPa for the sparse model, 574.46 MPa for the standard model, and 1569.04 MPa for the dense model, demonstrating a clear correlation between occluder density and stress magnitude (Figure 3A). In the standard configuration, peak stress was primarily localized at the connection zones. In contrast, the dense model exhibited a pronounced stress concentration, attributed to complete contact between the occluder and the crimping shell at a radial displacement of 6.75 mm. Beyond this point, continued compression led to excessive deformation caused by internal wire crowding, which in turn generated high localized stress within overstressed wire segments. Time-resolved stress evolution across all three configurations is illustrated in Figures 3C–E. The sparse model, characterized by a loose internal framework and wide inter-wire spacing, was less susceptible to compressive deformation. By contrast, the more compact architectures of the standard and dense models not only experienced higher external crimping forces but also substantial interwire interactions, further intensifying internal stress levels.

In all three occluder models, peak strain occurred at the point of maximum crimping. The recorded maximum strains were 1.88% for the sparse model, 2.73% for the standard model, and 12.73% for the dense model (Figure 3B). Both the sparse and standard configurations maintained strain levels well below the 8% superelastic recovery limit of nitinol, indicating that their structural integrity was preserved during compression. However, the dense model significantly exceeded this threshold with a peak strain of 12.73%, surpassing the commonly accepted recoverable strain limit for nitinol (Hassan et al., 2018). This suggests that overcompression in dense design may result in localized plastic deformation of the wire elements, potentially compromising the self-expansion capability.

**FIGURE 4:**
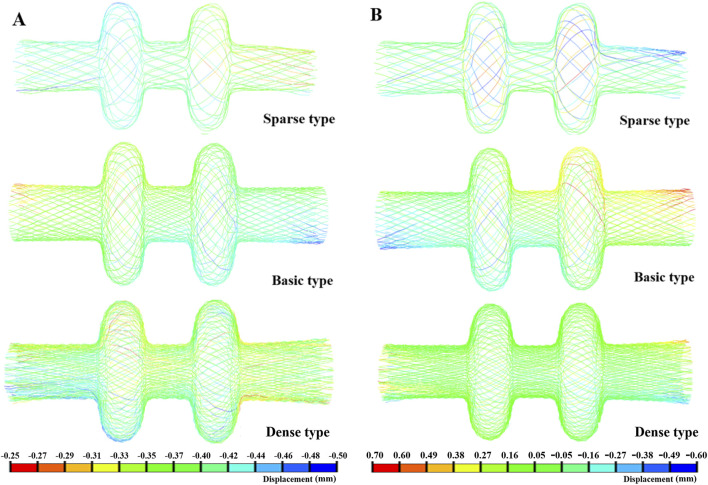
**(A)** Axial deformation; **(B)** Radial deformation.

As illustrated in Figure 4A, the sparse model experienced the greatest axial deformation, whereas the dense model the experienced least. The sparse occluder exhibited an axial shortening ratio of 2.83% (Table 4), indicating more pronounced longitudinal contraction during self-expansion and the weakest axial recovery among the three designs. The standard model demonstrated a moderate axial shortening of 1.83%, whereas the dense model had the smallest axial shortening at 0.94%, reflecting its superior axial resilience and minimal shape alteration following deployment. Radial deformation results are shown in Figure 4B. The maximum radial deformation measured was 0.084 mm for the sparse model, 0.081 mm for the standard model, and 0.077 mm for the dense model, showing relatively minor differences across configurations. Regarding radial recoil (Table 5), the sparse model had the highest recoil ratio at 1.05%, followed by the standard model at 0.81%, whereas the dense model exhibited the lowest radial recoil, only 0.15%.

**TABLE 4:** Axial shortening ratio ((L_1_-L_2_)/L_1_).

Models	Initial length $L_{1}$ (mm)	Expanded length $L_{2}$ (mm)	Axial shortening ratio (%)
Sparse	18	17.49	2.83
Standard	18	17.67	1.83
Dense	18	17.83	0.94

**TABLE 5:** Radial recoil ratio ((D_1_-D_2_)/D_1_).

Models	Initial diameter $D_{1}$ (mm)	Expanded diameter $D_{2}$ (mm)	Radial recoil ratio (%)
Sparse	8	8.084	1.05
Standard	8	8.081	1.01
Dense	8	8.077	0.96

### 3.2 Flexibility

**FIGURE 5:**
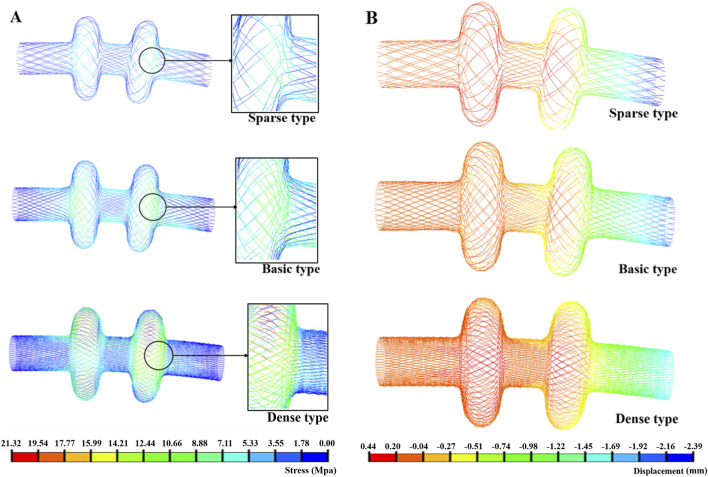
**(A)** Bending stress; **(B)** Bending deformation.

**FIGURE 6:**
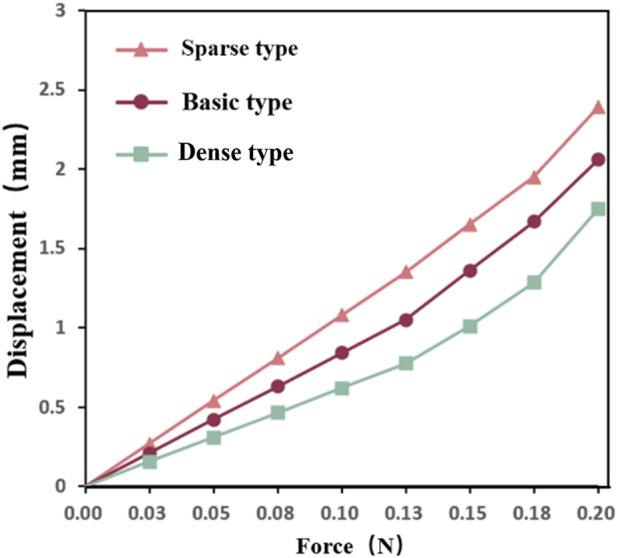
Bending displacement load curve.

In the flexibility tests, the dense occluder experienced the highest peak of von Mises stress at 21.32 MPa, while the sparse model exhibited the lowest stress of 8.26 MPa. The standard design fell between these values, reaching 12.91 MPa (Figure 5A). For all three configurations, stress concentrations were predominantly observed in the central and left connection zones. The corresponding maximum strain values were 0.22%, 0.14%, and 0.09% for the dense, standard, and sparse models, respectively—well below nitinol’s superelastic strain limit (Hassan et al., 2018). These findings indicate that all occluders can withstand bending without structural failure and maintain excellent elastic recovery. Analysis of maximum displacement revealed that the sparse occluder deformed more than the dense variant (Figure 5B). Additionally, displacement–load curves under bending (Figure 6) demonstrated that under identical loading, the sparse model produced the greatest deflection. Correspondingly, it had the lowest bending stiffness at 162.67 N/m (Table 6), reflecting superior flexibility. Conversely, the dense model showed the highest bending stiffness of 222.17 N/m, indicating reduced compliance. The standard occluder exhibited intermediate mechanical behavior, with a bending stiffness of 188.74 N/m.

**TABLE 6:** Bending stiffness of the occlusion devices ( $FL^{3}/3\delta$ , F , L , δ represent concentrated force, length of the occluder, and free-end vertical displacement, respectively).

Models	Length (mm)	Displacement (mm)	Bending stiffness (N/m)
Sparse	18	2.39	162.67
Standard	18	2.06	188.74
Dense	18	1.75	222.17

## 4 Discussion

Fallopian tube embolization represents a promising minimally invasive treatment for hydrosalpinx. However, existing occlusion devices suffer from notable shortcomings. For instance, the Essure® system, despite widespread use, has been linked to increasing reports of adverse events, largely attributed to its rigidity and sharp edges (Zou et al., 2023). In a study on the Essure® occluder (Hou, 2016), a case was reported where a 39-year-old woman underwent hysteroscopic sterilization with Essure. Although hysterosalpingography showed successful bilateral tubal occlusion, she subsequently experienced an unplanned pregnancy (which was terminated) along with persistent pelvic pain and other symptoms. Eventually, she underwent laparoscopically assisted vaginal hysterectomy and bilateral salpingectomy. Pathological evaluation revealed that one side of the fallopian tube was perforated by the Essure® occluder, while the fallopian tube on the other side (with the correctly placed occluder) remained patent.

The Essure® occluder consists of two metal coils, each covered with small plastic fiber barbs. After implantation into the fallopian tube, it induces scar tissue formation to achieve tubal occlusion. However, the fallopian tube itself undergoes physiological activities such as peristalsis, and the structural design of the occluder may not fully adapt to these physiological movements, making it prone to migration. If inserted not deep enough, the occluder may perforate the fallopian tube at the curved site and migrate into the abdominal cavity; if inserted insufficiently deep, the occluder may migrate into the uterus, causing severe spasms and heavy bleeding.

In addition, the inner coil of the Essure® occluder is coated with polyethylene terephthalate (PET) fibers. These fibers can stimulate a fibrotic response, which may lead to fallopian tube lumen occlusion (Djeffal et al., 2018). In an imaging study on mechanical fallopian tube occlusion devices (Guelfguat et al., 2012), the authors observed that migration of the Essure® coil device resulted in expulsion and other complications, with tubal expulsion into the uterine cavity being the most common complication of central migration. Similarly, the tornado-shaped microcoil suffers from structural instability due to its braided design, often necessitating multiple overlapping deployments and demonstrating a relatively high failure rate (Chen et al., 2022; Hochreiter-Hufford et al., 2024).

To overcome the problems that arise during the use of existing occluders, we developed a novel dual-layer spring occluder constructed from a shape-memory alloy, combining material and structural innovations. Compared to Essure® devices and tornado-shaped microcoils, due to the double-layer spring structure, the new occluder distributes stress more uniformly, reducing the risk of displacement. By adopting a more tightly woven structure, the stress on the structure can be more uniform, reducing the risk of puncture. By systematically varying the wire linear density as a single design parameter, we evaluated the mechanical behavior of different configurations through finite element analysis, focusing on crimping, deployment, and bending performance. Our simulations revealed that higher wire densities enhance radial support and elastic recoil but reduce compliance and increase the risk of material failure. Conversely, lower-density designs offer superior flexibility and safety but risk slippage due to their insufficient anchoring strength. Among the variants, the standard-density occluder achieved an optimal balance—delivering adequate support, effective recoil, and moderate compliance without inducing material damage—making it the most promising candidate for clinical application.

During the crimping and deployment stages, the distribution of peak stresses varied notably among the designs at maximum compression. In the sparse model, high stresses were concentrated in the support and central zones. The standard design exhibited stress predominantly at the connection points, while the dense occluder model lacked clear stress hotspots but showed localized concentrations in certain wire segments. This phenomenon resulted from excessive compression causing disorganized wire packing and over-deformation, with peak stresses reaching 1569.04 MPa. The sparse occluder, with its lower wire density, failed to achieve full contact with the crimping tool, relying mainly on the support zone for radial integrity, and accordingly showed stress concentration in those areas.

It is crucial to prioritize material safety in the design of occluders. Prior to this, several scholars had conducted research on the safety of NiTi materials. Maximilien E. Launey et al. (Launey et al., 2025) found that after NiTi materials underwent 100 million cycles under a maximum strain of 4%, there were no microcracks on the material surface, and no shift in the phase transition peak (determined by Differential Scanning Calorimetry, DSC). In fact, fallopian tube occluders only undergo a single crimping and release process during use. Within the range of maximum recoverable strain, the occluder can maintain structural and functional integrity. The maximum strains in both the sparse and standard models remained well below nitinol’s 8% superelastic strain limit (Hassan et al., 2018), indicating no permanent material damage during compression. The dense design, however, exceeded this threshold, suggesting localized plastic deformation and potential compromise of device function. Among the three, the standard occluder struck the best compromise, offering sufficient radial support to resist dislodgement within the smooth fallopian tubes while avoiding material failure. Although the dense occluder demonstrated marginally better axial shortening and radial recoil, these differences were minimal and unlikely to impact clinical outcomes significantly.

Load–displacement curves for all occluders displayed near-linear trends. Under identical loading, the sparse model showed the greatest deflection and lowest bending stiffness, indicating the highest flexibility. Conversely, the dense design was the stiffest, with minimal displacement, and the standard configuration exhibited intermediate mechanical behavior, balancing rigidity and compliance. Enhanced flexibility promotes easier navigation through the curved anatomy of delivery catheters and fallopian tubes, reducing risks of luminal abrasion or perforation. Thus, considering both flexibility and radial support, the standard occluder offers the most versatile and clinically applicable design (Siewert et al., 2023).

The biocompatibility of NiTi alloy can be better enhanced through approaches such as inhibiting nickel ion release, strengthening the surface passivation layer, and improving cytocompatibility and hemocompatibility. Nazarov D.V. et al. (Pawar et al., 2014) constructed a ZnO–TiO_2_ composite nanocoating on the NiTi surface via atomic layer deposition (ALD), which significantly promoted the adhesion and differentiation of osteoblasts (MG-63) and mesenchymal stem cells (FetMSC) while exhibiting a certain degree of antibacterial activity. Mohammadi et al. (2019) first performed anodization on NiTi, followed by coating with chitosan–heparin nanoparticles, which inhibited the release of Ni ions and improved anticoagulant performance.

Owing to its excellent metallic properties and biocompatibility, NiTi alloy is widely used in medical devices. Chen et al. (2016) developed a shunt for diverting blood flow away from aneurysm sacs, which is based on thin film nitinol (TFN). The results demonstrated that TFN exhibits excellent superelastic behavior, making it highly suitable for use in tortuous neurovascular arteries. Additionally, several studies on stents and occluders have also highlighted the advantages of NiTi alloy in terms of mechanics and biocompatibility (Elsisy et al., 2021).

Both ductility and biocompatibility are crucial properties required for fallopian tube occluders, and our novel occluder also adopts this material. Some occluders achieve occlusion by leveraging collagen tissue deposited in the submucosa during controlled fallopian tube inflammatory responses (Saad-Ganem et al., 2014). However, the occluder in this study achieves fallopian tube occlusion through its structure, and its favorable flexibility and material biocompatibility can prevent fallopian tube inflammation and device migration.

This study employed numerical simulations of crimping, deployment, and bending to comprehensively assess the mechanical performance of novel fallopian tube occluders with varying wire densities. The findings identify a standard-density design of the occluder that optimally balances radial support, elastic recoil, and compliance, offering a promising approach for hydrosalpinx treatment. However, future investigations should address the physiological responses of inflamed fallopian tubes under mechanical stimuli, as these may influence device efficacy. Additionally, compression enhances device compactness and potentially improves occlusion effectiveness; thus, further fluid dynamics studies are necessary to validate sealing performance *in vivo*.

## 5 Conclusion

This study introduces a novel shape-memory fallopian tube occluder fabricated from a NiTi alloy, featuring three geometric variants differentiated by wire density. Through numerical simulations evaluating crimping, deployment, and flexibility, distinct mechanical behaviors were observed across the designs. The sparse model (wire density = 84) demonstrated excellent flexibility but lacked adequate radial support. Conversely, the dense design (wire density = 226) provided enhanced structural strength but at the expense of compliance; overcrowding of internal wires caused excessive deformation surpassing the recoverable strain limit. The standard model (wire density = 113) achieved an optimal balance between mechanical support and structural resilience, highlighting its strong potential for clinical use.

## Data Availability

The raw data supporting the conclusions of this article will be made available by the authors, without undue reservation.
